# Antimicrobial Polymers for Additive Manufacturing

**DOI:** 10.3390/ijms20051210

**Published:** 2019-03-10

**Authors:** Carmen Mabel González-Henríquez, Mauricio A. Sarabia-Vallejos, Juan Rodríguez Hernandez

**Affiliations:** 1 Departamento de Química, Facultad de Ciencias Naturales, Matemáticas y del Medio Ambiente, Universidad Tecnológica Metropolitana, Las Palmeras 3360, Santiago 7800003, Chile; 2 Programa Institucional de Fomento a la Investigación, Desarrollo e Innovación, Universidad Tecnológica Metropolitana, Ignacio Valdivieso 2409, Santiago 8940577, Chile; 3 Departamento de Ingeniería Estructural y Geotecnia, Escuela de Ingeniería, Pontificia Universidad Católica de Chile, Avenida Vicuña Mackenna 4860, Santiago 7820436, Chile; masarabi@puc.cl; 4 Instituto de Ingeniería Biológica y Médica, Pontificia Universidad Católica de Chile, Avenida Vicuña Mackenna 4860, Santiago 7820436, Chile; 5 Polymer Functionalization Group, Departamento de Química Macromolecular Aplicada, Instituto de Ciencia y Tecnología de Polímeros-Consejo Superior de Investigaciones Científicas (ICTP-CSIC), Juan de la Cierva 3, 28006 Madrid, Spain

**Keywords:** additive manufacturing, antibacterial polymers, biocompatible systems, drug delivery systems, 3D printing

## Abstract

Three-dimensional (3D) printing technologies can be widely used for producing detailed geometries based on individual and particular demands. Some applications are related to the production of personalized devices, implants (orthopedic and dental), drug dosage forms (antibacterial, immunosuppressive, anti-inflammatory, etc.), or 3D implants that contain active pharmaceutical treatments, which favor cellular proliferation and tissue regeneration. This review is focused on the generation of 3D printed polymer-based objects that present antibacterial properties. Two main different alternatives of obtaining these 3D printed objects are fully described, which employ different polymer sources. The first one uses natural polymers that, in some cases, already exhibit intrinsic antibacterial capacities. The second alternative involves the use of synthetic polymers, and thus takes advantage of polymers with antimicrobial functional groups, as well as alternative strategies based on the modification of the surface of polymers or the elaboration of composite materials through adding certain antibacterial agents or incorporating different drugs into the polymeric matrix.

## 1. Introduction

According to the International Organization for Standardization/American Society for Testing and Materials (ISO/ASTM) standards (from a report of Technical Committee F42), additive manufacturing (AM) is defined as “the process of joining materials to make parts from 3D model data, usually, layer by layer” [1]. In the last years, several advances have been performed in AM technologies by the scientific community, thus generating an abrupt and quick evolution in the development of these methods. As mentioned by Campbell and Ivanova [2], AM is today widely considered a disruptive technology that offers a new paradigm for engineering design and manufacturing that could have significant economic, geopolitical, environmental, intellectual property, and security implications. 

The use of additive manufacturing (AM) methods, which are commonly known as three-dimensional (3D) printing, has allowed the fabrication of an endless variety of devices with a wide myriad of potential applications in several fields, and particularly in the bioengineering area [3,4]. 3D printing methodologies permitted us, for instance, to manufacture or fabricate structures with architectures closely similar to biological tissues, such as bones, cartilages, or heart valves. With AM technology, complex geometrical cues with high accuracy can be straightforwardly achieved. Moreover, the increasing demands and applications of tissue engineering, antimicrobial/anti-biofouling devices, and regenerative medicine, researchers are seeking new manufacturing technologies that could solve the supply shortage of tissues or organs and the immunological requirements of implanted devices [5]. The fields of biomedicine, food fabrication, packaging, paintings, and naval [6], among others, have not been the exception. For example, there are several examples of polymeric-based biomedical devices, including artificial hips and knee implants or systems such as stents, heart valves, or even vascular grafts, which are commonly used both to improve the quality of life and, in some cases, increase life expectancy.

In spite of this, a still remaining fundamental problem in the employment of polymeric materials for biomedical purposes is related to material contamination by a wide variety of microorganisms. This issue is nowadays a critical limitation for the use of polymeric materials in this application field. Particularly, in the biomedical area, this topic is important because it is highly possible that bacteria could be present in the media, affecting the integrity of the medical devices and healthcare products [7]. 

Another common problem associated with contamination by microorganisms, which needs to be solved in a short time, is the biofilm formation over 3D printed structures [8]. Understanding the relationship between the extracellular matrix and the 3D topography of the material could be fundamental for describing the mechanisms of matrix formation, mechanosensing, matrix remodeling, and the modulation of cell–cell or cell–matrix interactions during biofilm formation [9], which would allow one more step toward the solution for avoiding biofilm formation.

In this context, this short review will attempt to briefly describe the most relevant and recent advances in the elaboration of antimicrobial 3D printed devices and objects. We will first briefly describe the principles of additive manufacturing and the basics of antimicrobial polymers. The following sections will be devoted to the description of illustrative examples of the different strategies that have been reported for the fabrication of antimicrobial objects using natural occurring polymers, but also using synthetic polymer blends with antimicrobial agents.

## 2. Principles of Additive Manufacturing

**Figure 1:**
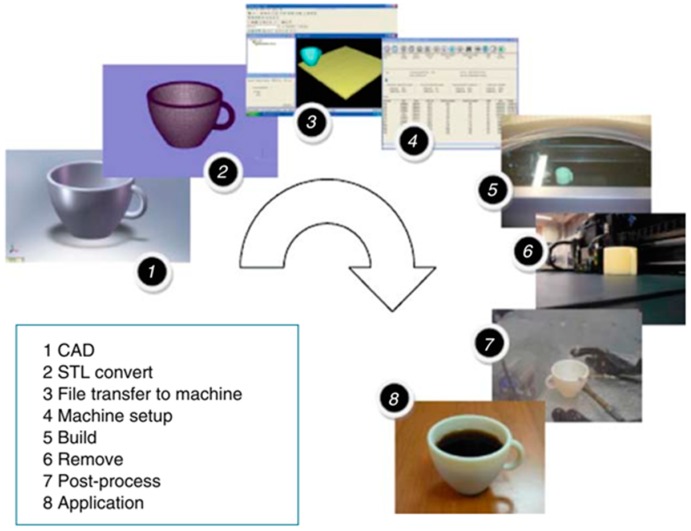
Steps involved in the fabrication of a cup by additive manufacturing (AM). Reproduced with permission from Ref. [1].

As described in Figure 1, AM starts with the design of a three-dimensional (3D) model (Step 1), which is then transferred to the printing machine (steps 2 to 3). Then, the model is digitalized and sliced into several layers. Set-up parameters are introduced (Step 4), including the energy source (temperature, laser intensity, etc.) or z-resolution (provided by the layer thickness). Once the experimental conditions are selected, the AM system prints the layers in a build, adding each new layer on top of the prior one (Step 5). The final steps involve post-processing to remove the supporting material (steps 6 to 7), and finally, its use for the application for which it was designed (Step 8).

There are several types of AM methodologies; the most common types are the ones based on material extrusion, such as fused deposition modeling (FDM) or bio-plotting [10], which are particularly interesting for this review, because it enables manufacturing parts with several types of biocompatible or biodegradable polymers, and in some cases, with living cells or bacteria [11]. Other common technologies are the stereolithography (SLA), technologies based on partially-melting powder such as selective laser sintering (SLS), and full-melting powder such as selective laser melting (SLM) or binder jetting. 

Additive manufacturing offers important advantages over other currently employed technologies for prototyping [12]. AM permits the fabrication of fully customized geometrically complex products in an economic manner for limited production. Some reports established that AM is cost-effective in comparison, for instance, with plastic injection molding for targeted production runs ranging from 50 to 5000 units. Other authors estimated that AM is competitive with plastic injection molding for the targeted fabrications below 1000 items [13]. The basis of the low production cost is related to not needing molds or costly tools, there being no requirements for milling or sanding processes, and the full automatization of the process. 

Another crucial advantage is related to the design of the AM printed parts. Designs can be easily created and modified according to any required change, and can be shared so that manufacturing can be easily carried out in many different places simultaneously. In fact, AM allows for the quick fabrication of prototypes with different versions for lab testing without the need for costly retooling. Moreover, replacement parts can be produced by third-party providers utilizing the original designs provided by the manufacturer. As a result, an inventory is not required that can suppose additional costs if finished goods remain unsold.

Finally, it is worth mentioning that AM offers important improvements in terms of environmental implications, because it results in an efficient use of the materials and permits an environment-friendly design.

**Figure 2:**
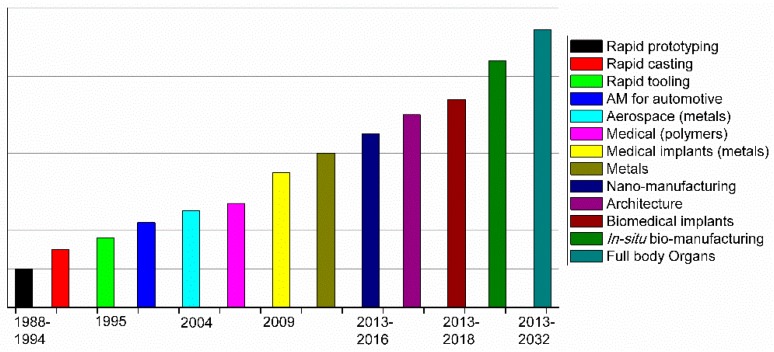
AM timeline for different applications. Reproduced with permission from Ref. [14].

As shown in Figure 2, which was extracted from Mawale et al. [14], a clear evolution from rapid prototyping to series production can be observed in the latest years. The future of AM should be focused, according to Mawale et al., on the efficiency of AM processes to decrease the final fabrication time and price.

In terms of applications, the future still requires further investigations in areas of biomedical devices, in situ biomanufacturing, or in the fabrication of full body organs. AM technologies have been steadily growing during the last 20 years, and today, AM parts accomplish the requirements of many different industrial areas, particularly in biomedical applications including implants/prosthetics, dental, and surgical devices/aids [12,15]. Some of these devices use ceramics or bioceramics as a base for 3D printing [16], but in this review, we will be focused on the usage of polymeric materials (or some polymeric composites) for fabricating antimicrobial devices and objects.

A crucial disadvantage for bio-related applications that needs to be solved quickly for some of the materials that are used in AM methodologies is their natural cytotoxicity. Zhu et al. showed that several SLA resins are highly toxic, impeding the developing of zebrafish embryos [17]. The authors have postulated that chip-based devices manufactured using SLA processes leached toxic chemicals to the culture media, causing significant toxicity, as evidenced by the highly sensitive zebrafish embryo toxicity biotest [17]. Other groups focused their studies on the potential chemical hazard profiles and limitations associated with the fabrication of biocompatible devices using SLA, FDM, and multi-jet modeling (MJM) processes [18]. Here, three distinctive biotests were selected for testing the potential chemical hazard risks associated with 3D printed polymer leachates. These results show that the majority of SLA and MJM polymers exhibit toxic effects, which is a problem for biological applications. Table 1 contains a summary of the toxicity data available for some of the composites that are used in SLA printing.

## 3. Innovative AM Technologies

To carry out a deep discussion about the antimicrobial polymers used in AM methodologies, it is important to first mention some of the most common types and innovative AM technologies. The ASTM technical committee F42 define the criteria that has been employed for classifying the AM technologies into seven different categories: (a) material extrusion, (b) powder bed fusion, (c) vat photopolymerization, (d) material jetting, (e) binder jetting, (f) sheet lamination, and (g) directed energy deposition [40,41]. While it is true that today there exists a wide variety of materials that are used in AM including thermoplastics, photopolymers, ceramics, and metallic powders, we will center our attention on those techniques that exclusively employ polymeric materials and composites, and particularly those that use antimicrobial polymers for biomedical or biotechnological applications. Among these methods, material extrusion, powder bed fusion, and vat photopolymerization are the most common techniques to create 3D printed devices, and consequently, they have been extensively employed in conjunction with polymeric antimicrobial composites. 

There are also some innovative technologies that are based on one or more of the previously mentioned technologies. A novel methodology is for example that reported by Chang et al. [42], who developed a technique for 3D printing that was coined electrohydrodynamic printing (EHD), which is based on the application of a controlled electric current through a syringe that follows a predetermined path layer-by-layer to form a 3D printing part. Another interesting example is the reported by Malinauskas et al. [43] from the year 2017, which developed an ultrafast laser lithography methodology by using a femtosecond laser to create micro-optical devices (lenses) with optical clearness and high resilence. Additionally, they proved a controlled pyrolysis procedure as a method to shrink the printed parts in order to achieve smaller dimensions without compromising the resolution of the printed part. Malinauskas et al. achieved a 40% volumetric shrinkage by removing the organic constituents of their printed parts via pyrolysis. 

Similarly, the group of Jeon et al. [44] developed a method coined high-resolution 3D interference printing, which is based on the optical interference phenomenon with restricted near-field diffraction generated by conformal binary gratings. The advantage of this methodology is that it involves only one single ultrashort exposure step. The design of the conformal grating allows controlling the geometry of the printed parts, permitting obtaining high-resolution printing devices in a short amount of time. Another similar methodology that allows obtaining high-resolution devices of multiple materials in a single exposure step is that coined by the group of Hawker et al. [45] as Solution Mask Liquid Lithography (SMaLL). In this case, photochromic molecules are used to control the polymerization kinetics through coherent bleaching fronts, providing large curing depths and rapid build rates without the need for moving parts. The coupling of these photoswitches with some resin mixtures allows the simultaneous and selective curing of multiple networks, providing access to 3D printed parts with chemically and mechanically distinct domains. 

The group of Watanabe et al. [46] reported an innovative method for 3D printing based on a plasma ion beam. They used a high-current plasma focused ion beam (FIB) to generate customized microelectromechanical systems (MEMS). This methodology allows creating capacitive MEMS vibration sensors, which were compared to their counterparts fabricated with conventional lithography methods. The difference in measured resonance frequency was small (less than 4%), but the fabrication times were reduced by more than 80% using this methodology.

There are some innovative printing method that are not directly linked with the topic of this review (antimicrobial 3D printing), but deserve to be mentioned due to their originality and novel applications; one of these is the so-called 3D ice printing [47] developed by the group of Zhang et al., which uses this technique to print microcapsule chip arrays over different substrates for target detection. This printing method allows storing the microcapsule arrays in ice form before use, guaranteeing their long-term stability. Other methods, such as that reported by Shear et al. [48], enable 3D printing bacterial communities that are encapsulated in polypeptide matrices of particular and complex shapes and forms. In this article, the authors described a strategy that enabled printing multiple bacteria populations that could be organized within essentially any 3D geometry, including adjacent, nested, and free-floating colonies. The printing method works with a laser-based lithographic technique that permits the formation of microscopic containers around selected bacteria suspended in gelatin via focal cross-linking of the after-mentioned polypeptide molecules.

## 4. Antibacterial Polymers: Few Elementary Aspects

Pioneer studies on antimicrobial molecules have been mainly focused on the use of low molecular weight substances. However, Boman [49] et al. discovered that a particular family of peptides, i.e., antimicrobial peptides (AMPs), were excellent candidates to remove gram-positive and gram-negative bacteria, as well as also fungi. Since these initial reports AMPs have been extensively investigated, and a large amount of peptides have been reported, classified, and gathered in an antimicrobial peptide database (APD) [50]. The investigations that were carried out at that time concluded that a major part of the AMPs presented a common characteristic, i.e., they were formed by a combination of amino acids with polar and non-polar side chains, thus leading to amphiphilic structures. The polar units are typically formed by amino acids such as lysine or glutamic acid, and the non-polar parts are formed by the incorporation of amino acids such as tryptophan. This amphiphilic characteristic has been demonstrated to play a key role in the interaction of the AMPs with the bacterial membrane, and was employed as a starting point to develop antimicrobial polymers mimicking this polar/non-polar characteristic. [51] As a result, a variety of synthetic polymers with variable chemical structures and functional groups have been employed for the preparation of antimicrobial macromolecules, in some cases with effective antimicrobial activity. [52,53,54,55,56,57] In this section, we aim to briefly summarize the most relevant characteristics of the antimicrobial polymers reported, taking into account their structure and functionality.

### 4.1. Polymer Requirements: Polymeric Structures and Other Relevant Characteristics

Matsuzaki [58] proposed a list of four major features that an antimicrobial polymer should have in order to exhibit efficient antimicrobial activity. These features are based on the analysis of the structure of the bacterial membrane and the mechanisms involved in the disruption of this membrane. The four major characteristics are:

(a) able to establish enough contact with the microorganisms,
(b) the polymer should have enough cationic groups so that the adhesion to the microorganism cell can occur,
(c) the polymer should also be designed with hydrophobic moieties that are responsible for the attachment and integration inside the cellular membrane.
(d) the polymer must selectively kill the microbes in the presence of other cells such as mammalian cells.

These initial four major characteristics were complemented with another proposed by Kenawy et al. [7], taking into account the antibacterial mechanism and the synthetic aspects involved in the fabrication of the polymer. Then, these authors proposed that a model antimicrobial polymer should also:

(1) be synthesized using easy and cost-effective strategies
(2) be stable for the applications that can in some cases require long-term usage and storage at a certain temperature
(3) the polymer should remain insoluble in aqueous solution
(4) not decompose or release toxic products, and should not be toxic or irritating to users
(5) ideally be regenerated, maintaining its activity, and finally,
(6) be able to target different pathogenic microorganisms in a relatively short period of time.

### 4.2. Types of Antimicrobial Groups Integrated in Polymers

From a synthetic point of view, several aspects can be tailored for the synthesis of antimicrobial polymers. Herein, we will briefly discuss these aspects, which are divided in two main groups. On the one hand, the functional groups that were inserted in the polymer structure play a key role in the interaction with the microorganism membrane. On the other hand, those polymer characteristics are related with the structure of the polymer, including the molecular weight and distribution of the functional groups. 

For instance, it is today widely admitted that bacterial cell membranes (both gram positive and gram negative) are formed by phospholipids and teichoic acids with a negative net surface charge, among other materials. Therefore, polymers bearing positively charged functional groups are in principle able to interact better with the bacterial cell wall in comparison to neutral or negatively charged functional groups. 

#### 4.2.1. Positively Charged Functional Groups

Without any doubt, quaternary ammonium/phosphonium groups are the most extensively employed functional groups for the preparation of cationic polymers and have been reported to act as efficient antimicrobial agents. 

Pioneer works using ammonium chlorides were reported in the early 1980s by Ikeda et al. [59,60], which described the preparation of polyvinylbenzyl ammonium chloride. In their studies, Ikeda et al. evidenced high antimicrobial activity, and associated this activity with the possibility of interaction between the polymer and the cell membrane [61]. In addition to the above-depicted quaternary amine functional groups, other functional groups that are able to be protonated, depending on the environmental pH, have been equally reported. In this context, primary, secondary, and tertiary amino groups have been also explored. An illustrative example was reported by Gelman et al. [62]. This group synthesized a wide range of polymers based on polystyrene comprising tertiary amine groups. After the protonation of the tertiary amino groups, the authors observed an activity similar to that exhibited for quaternary amine functional groups. Dimethylamino ethyl methacrylate (DMAEMA) is a monomer with tertiary amino side functional groups that can be protonated when in contact with humidity or in aqueous media. This monomer was employed by Vigliotta et al. [63] to prepare a wide range of polymers based on DMAEMA that resulted in materials with excellent antimicrobial activity.

While the above-depicted functional groups are reported to have excellent antimicrobial activity, they present a major drawback: the toxicity against mammalian cells. In order to reduce this undesirable side effect, several groups have worked on the use of alternative cationic groups. In this context, phosphonium groups were demonstrated to be less toxic to mammalian cells, which together with an improved thermal stability, have been proposed as candidates for the incorporation in the elaboration of antimicrobial polymers [64,65]. Dehelean et al. [66], Ao et al. [67], and Zhao et al. [68] employed this type of functionality for the preparation of different antimicrobial polymers. For instance, Ao et al. [67] employed quaternary phosphonium groups to modify epoxy natural rubber, and evidenced an improved antibacterial activity of these materials in comparison to the non-modified ones. Also, Zhao et al. [68] employed quaternary phosphonium groups, but for the fabrication of terpolymers also bearing polyacrylamide. Interestingly, the authors evidenced an excellent activity in particular for adenovirus (ADV). Furthermore, the authors showed that the antibacterial activity is directly related to the amount of quaternary phosphonium within the polymer. 

#### 4.2.2. Other Antimicrobial Functional Groups

In addition to the cationic quaternary ammonium and phosphonium groups, *N*-halamine groups [69,70], sulfonium [71], zwitterionic polymers [72], and nitric oxide-containing polymers [73] are a few examples of the groups with excellent antimicrobial activity.

For instance, *N*-halamines are interesting candidates that have been extensively studied during the last decade, and have been reported as having a large activity against a wide variety of microorganisms while also being safe for the environment and humans. *N*-halamines can be regenerated, and can be produced at low cost. [69] According to Hui et al. [74], *N*-halamines have been incorporated in polymers by using different alternatives that include the polymerization approach (homopolymerization and heteropolymerization), the electrogeneration of biocidal coatings, or by grafting or coating on different substrates (typically by using epoxides, hydroxyl groups, and alkoxy silanes as anchoring groups). 

**Figure 3:**
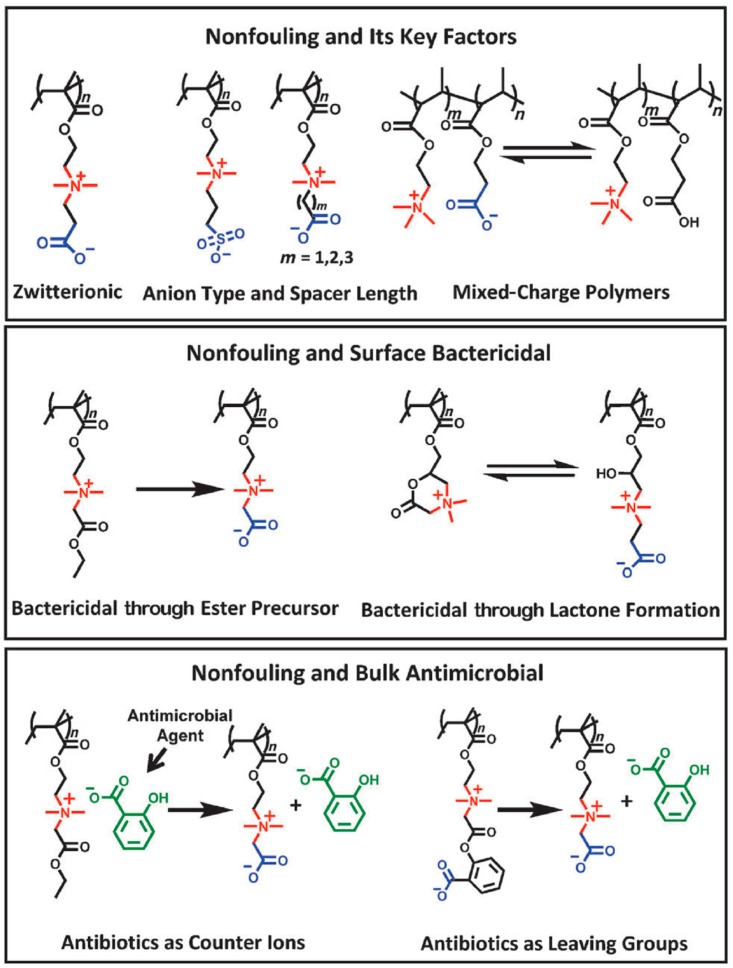
Chemical structures and microbiological applications of several zwitterionic polymers and their derivatives. Reproduced with permission from Ref. [75].

**Figure 4:**
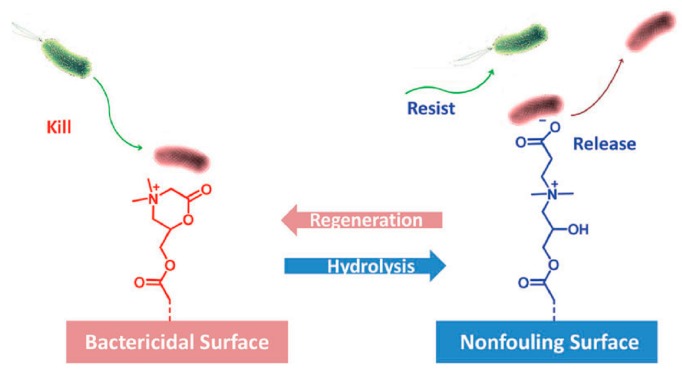
Illustrative scheme of the reversible lactonization that enables the system to kill bacteria (cationic state) and the release of inactivated bacterial cells occurring upon ring-opening and formation of the zwitterionic state. Reproduced with permission from Ref. [75].

Another example is the case of zwitterionic polymers. These groups present simultaneously negative and positively charged groups that result in a net neutral charge. Illustrative studies on the use of these functional groups were reported by Lowe et al. [72,73]. Jiang et al. [75] investigated a large number of these polymers, and they found some of them to present excellent antimicrobial activities in particular toward *E. Coli.* and *S. Aureus*. In a recent minireview, Jiang et al. summarized the capabilities of zwitterionic polymers as antimicrobial and simultaneously non-fouling groups [75]. They reported the recent advances of the different chemical structures of zwitterionic polymers and divided the groups into functional types depending on the microbiological application, i.e., non-fouling, non-fouling and surface bactericidal, and non-fouling and bulk antimicrobial (Figure 3). A particularly interesting example is the development of polymers that are able to kill, release, and be regenerated to restore the antimicrobial activity. In effect, for many purposes, such as reusable medical devices, the recovery of the bactericidal activity is needed. In many applications, such as most reusable surgical devices, renewable bactericidal surfaces are often needed. For this purpose, Cao et al. designed zwitterionic polymers that have the ability to undergo a reversible lactonization reaction that served to create reversible cycles between bactericidal and non-fouling states (Figure 4) [76,77].

### 4.3. Macromolecular Characteristics and their Role in the Antibacterial Activity

In addition to the chemical functional groups, several other parameters related to the polymer design play an important role. It is outside of the scope of this review to provide a detailed description of all the aspects involved; instead, we will just focus on briefly describing the most relevant characteristics, which include the hydrophilic/hydrophobic balance, the molecular weight, or the polymer topology (block/random copolymers or branched/linear polymers).

Without any doubt, one of the most critical aspects is the amphiphilicity of the polymer, i.e., the hydrophobic/hydrophilic balance. The amphiphilic character affects both the antimicrobial activity as well as the selectivity toward mammalian cells. An ideal amphiphilic polymer is formed by hydrophilic moieties, which are typically positively charged groups, and hydrophobic moieties (usually alkyl chains) forming the main polymer chain [78]. The design of this polymeric structure is based on two main ideas. First of all, it provides electrostatic interactions between the negatively charged cell membrane and the cationic groups in the polymers. Second, the hydrophobic segments within the polymer chain will be able to establish interactions with the lipid domains within the cell membrane.

As a result of this configuration, the negatively charged cell membrane will interact with the positively charge moieties and the hydrophobic main chain will be in contact with the lipid domains of the membrane [79]. However, an appropriate balance has appeared to be crucial. For instance, it has been reported that large hydrophilic moieties bind better to the cell membrane. While this is true for the hydrophilic segment, it has been proved that too large hydrophobic segments result in an increase of the cytotoxicity for all cell types [80].

Also, the molecular weight of the polymer plays a key role in both the antimicrobial activity as well as the hemolytic activity; however, it is worth mentioning that, in this case, the results that are reported largely depend on the type of cells employed and the type of polymer. In principle, the interaction between the polymer and the cell wall depend on the positive charges within the polymer structure; it is expected that higher molecular weight polymers provide better antimicrobial activities in comparison to small-sized polymers or oligomers. Nevertheless, high molecular weight polymers present important limitations such as their solubility or eventual aggregation in biological medium, but more importantly, increasing hemolytic activity (directly related to the polymer size).

Finally, also, the polymer topology significantly affects the antimicrobial activity of the polymer. Since the arrangement of polar and non-polar groups within the macromolecular structure affects the antimicrobial activity, the behavior of block copolymers and random copolymers can be significantly different. In general, random copolymers present rather good antimicrobial activities, but lack the required selectivity [81], although some studies have used alternative monomers that can be activated in acid conditions but have little hemolytic activity at neutral pH [81], or used long hydrophilic and cationic polymers [82]. In the case of amphiphilic block copolymers, they present the unique capability of forming different types of nano-objects in solution by self-assembly. The morphologies that they can form depend on the hydrophilic to hydrophobic volume ratio, but also on the molecular weight, as well as other experimental conditions such as the temperature or solvent employed. While it is true that there are not many examples in the literature, some reports indicated that the block copolymers are much less hemolytic compared to the highly hemolytic random copolymers. In an interesting manuscript, Oda et al. [83] compared the antimicrobial activity and the hemolytic activity of copolymers based on isobutyl vinyl ether (IBVE) and phthalimide-protected amine vinyl ether (PIVE) with similar chain lengths and monomer compositions. The block copolymers displayed selective activity against E. coli over red blood cells (RBCs), while the random copolymers did not and were hemolytic.

## 5. Antimicrobial Polymers, Blends, and Composites in Additive Manufacturing

Antimicrobial 3D printed parts have been intensively investigated in recent years due to the wide range of applications, including bone tissue engineering regeneration [84,85] to treat bone fractures [86], the fabrication of biomedical devices that are able to prevent biofilm formation [8], the fabrication of wound dressings [87], or the fabrication of scaffolds, just to mention few of them [88].

Independently of the application, in order to provide antimicrobial or antibacterial activity to a 3D printed part, different alternative strategies can be employed, which include among others the use of antimicrobial polymeric materials, the incorporation and release of antimicrobial agents, or introducing an antibacterial functionality through the surface modification of the part [89]. As will be depicted, both natural and synthetic polymers have been employed to fabricate antimicrobial 3D pritned parts. Moreover, not only polymeric but also composite materials, i.e., raw materials that present antibacterial characteristics or polymeric composites that are mixed with bactericide compounds to create antibacterial materials, have been reported. 

In the following sections, we will describe the fabrication of different antimicrobial 3D printed parts depending on the nature of the polymer employed (natural or synthetic). In Section 5 and Section 6, we attempt to differentiate among the alternative approaches that have been depicted to fabricate the parts such as the incorporation of antimicrobial monomers, antimicrobial agents (non-polymeric/monomeric), and surface modification. The following Section 7 will be focused on the description of those systems based on polymer composites with antimicrobial properties.

## 6. Naturally Occurring Antimicrobial Polymers

**Figure 5:**
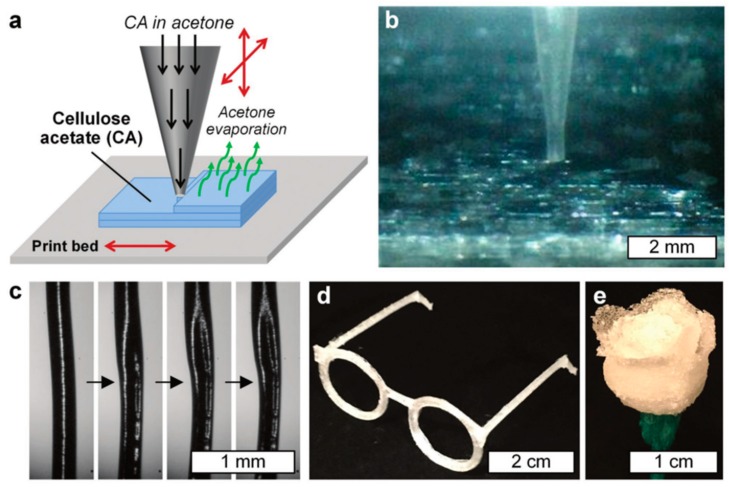
(**a**) Schematic description of the three-dimensional (3D) printing process using cellulose acetate (CA) dissolved in acetone. (**b**) Photograph of a close-up of printing tip during the manufacturing process. (**c**) Micrographs that show the acetone evaporating process. 3D printed parts, (**d**) eyeglass frames, and (**e**) a rose. Reproduced with permission from Ref. [92].

The use of naturally occurring polymers for AM applications has been increasing in recent years. In this context, without any doubt, cellulose—and most recently nanocellulose—is the most extensively employed natural type of polymer due to its intrinsic antimicrobial properties. The rationale behind this selection relies on cellulose being one of the primary reinforcement structures of most of the biological organisms, and indeed, it is one of the most abundant polymers on Earth [90]. Cellulose is also mechanically robust, inexpensive, biorenewable, biodegradable, chemically versatile, and also has antimicrobial properties [91]. All of these characteristics make this polymer considerably attractive for use in AM technologies, and for biomedical applications in particular. For instance, Pattinson and Hart [92] reported a method for printing a cellulose-based material via the extrusion of cellulose acetate (CA). The CA feedstock was prepared by dissolving it in acetone; then, the extrusion was performed via a gantry-style 3D printer with a capillary nozzle connected to a liquid dispenser. Once the material was extruded from the tip, the acetone evaporated, thus leaving a CA printed layer-by-layer device. In their work, Pattinson and Hart, changed the printing parameters such as liquid pressure/flow, print velocity, and distance from tip to the print bed, and solution viscosity. Figure 5a shows a schematic description of the CA printing process. Figure 5b shows a close-up of the printing process.

**Figure 6:**
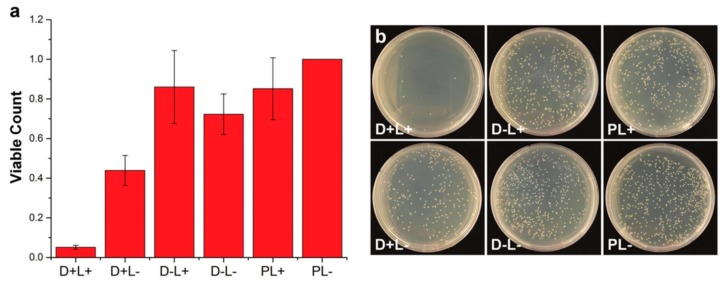
(**a**) Evaluation of the antimicrobial performance of the 3D printed CA parts, in which the viable bacteria count was normalized with the PL- results; and (**b**) Images of the bacteria cultured Petri dishes exposed to different conditions. Adapted with permission from Ref. [92].

Uniaxial tests of CA printed parts were carried out to test the mechanical properties of the resulting parts. For that purpose, dogbone samples, which were printed at different printing directions (parallel and perpendicular) and with variable material treatments (for instance, with NaOH), were mechanically tested. The results demonstrated a favorable mechanical performance compared with other common thermoplastic AM materials (Acrylonitrile Butadiene Styrene (ABS), poly(lactic acid) (PLA), and nylon). The NaOH treatment effectively transforms the CA into cellulose via a deacetylation process, as evidenced by Fourier transform infrared (FTIR). It is important to remark that the mechanical behavior of the CA probes is not considerably altered. Finally, the antimicrobial effectivity of the printed parts was tested using *E. coli*. To compare the results, small amounts of known antimicrobial agent dyes (toluidine blue and rose bengal) were added to Petri dishes with the CA printed material and exposed to a fluorescent lamp; additionally, polyethylene substrates were used as a control. Different set-ups were tested, which were namely as follows. D+L+: Bacteria exposed to cellulose acetate with dye and light. D+L−: Bacteria exposed to cellulose acetate with dye, but left in the dark. D−L+: Bacteria exposed to cellulose acetate without dye, but with light. D−L−: Bacteria exposed to cellulose acetate with no dye and left in the dark. PL+: Bacteria exposed to polyethylene and light. PL−: Bacteria exposed to polyethylene and left in the dark. Figure 6 shows some of the results obtained from viable bacteria count. This data demonstrates that the dyed CA printed parts enabled a large reduction in the bacterial count by simple exposure to fluorescent light. 

**Figure 7:**
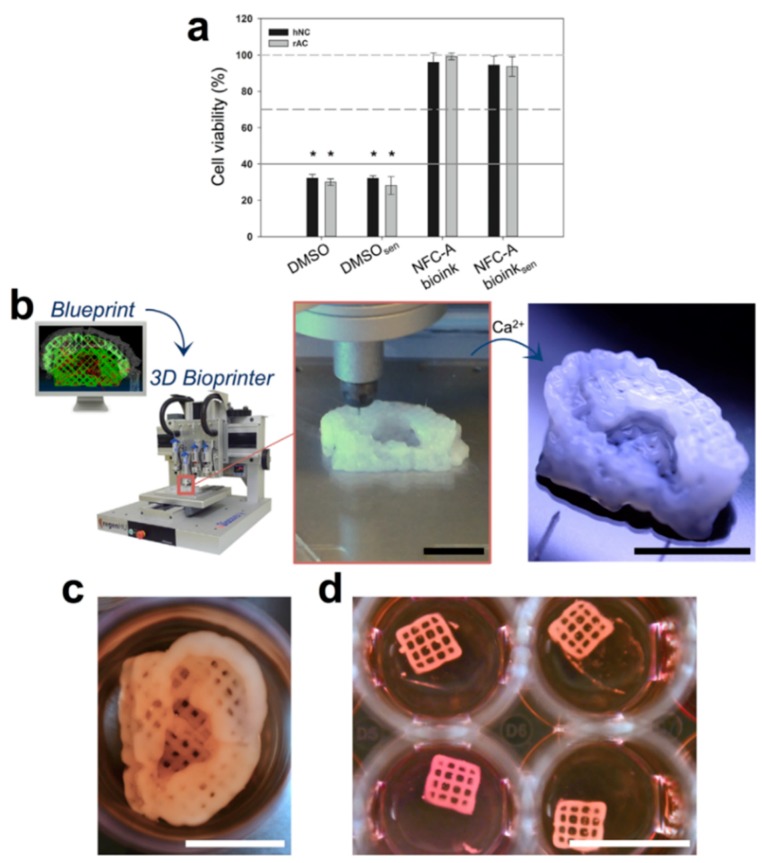
(**a**) In vitro cytotoxicity test of Nanofibrillated cellulose (NFC) bioink. Human nasal chondrocytes (hNC) and rabbit auricular chondrocytes (rAC) were used as indicator cells to determine the cytotoxic effects potentially caused by bioink components, cross-linking, or the bioprinting process. (**b**) 3D bioprinting process of chondrocyte-laden NFC-A auricular construct with open porosity. (**c**) 3D bioprinted auricular and (**d**) lattice-structured constructs, laden with hNCs, after 28 days of culture. Reproduced with permission from Ref. [93].

Recently, Gatenholm et al. [93] reported an innovative 3D printing method based on nanocellulose hydrogels aiming to provide an alternative treatment to serious auricular defects. A matrix phase of alginate mixed with nanofibrillated cellulose particles was cross-linked by using a CaCl_2_ solution (Figure 7b). Human nasal chondrocytes (hNC) were mixed with the ink to obtain the bioink used for print (20 × 10^6^ cells/mL), and then were printed into auricular constructs with open porosity (Figure 7c). Cell viability was examined at different stages of the bioprinting process: before embedding the hNCs, before embedding and cross-linking, and after embedding, bioprinting, and cross-linking. The mean cell viability after the embedding and cross-linking processes was found to be significantly lower than the samples before embedding the hNCs (Figure 7a). Furthermore, no significant difference between after embedding and after bioprinting was founded, indicating that the printing process had no significant influence on cell vitality. Based on these results, it was concluded that this bioink is promising for auricular cartilage tissue engineering and many other biomedical applications.

## 7. Synthetic Polymers with Bactericidal Properties

### 7.1. Antimicrobial Functional Monomers as Components in SLA Resins

**Figure 8:**
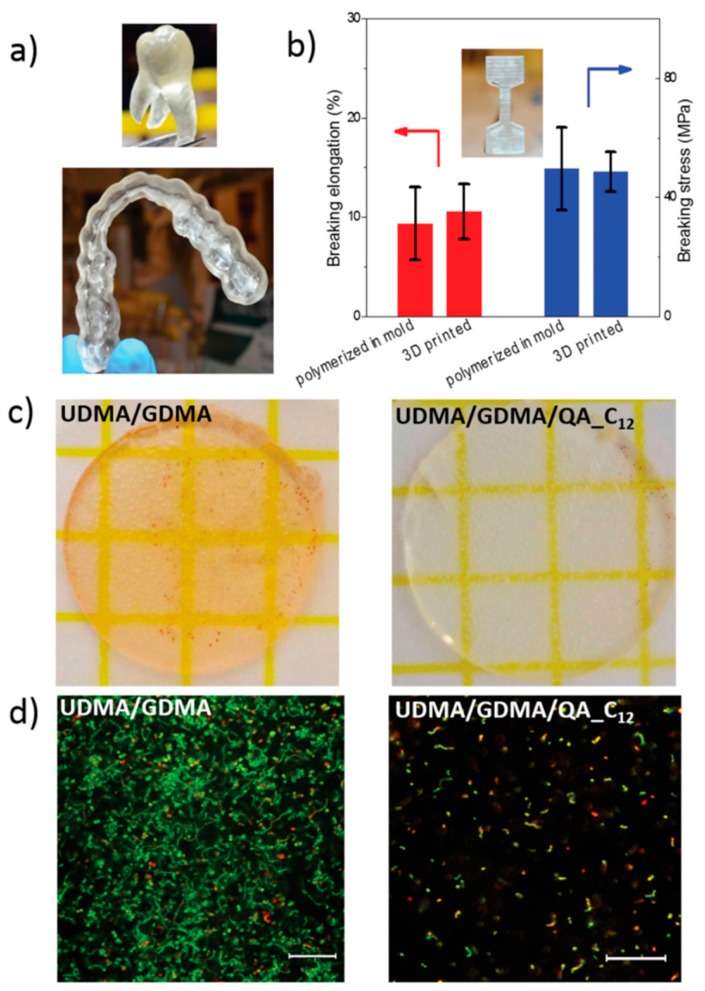
(**a**) 3D printed dental parts using diurethanedimethacrylate/glycerol dimethacrylate (UDMA/GDMA) composites. (**b**) Uniaxial tensile tests of 14 mol% UDMA/GDMA with the modified methacrylate monomers with an alkyl chain length of *n* = 12 (QA_C_12_). (**c**) Comparison of the contact-killing efficacy of 3D printed UDMA/GDMA and UDMA/GDMA/QA_C_12_ and (**d**) comparison of the long-term contact-killing efficacy of 3D printed UDMA/GDMA and UDMA/GDMA/QA_C_12_ six days after live/dead staining. Reproduced with permission from Ref. [94].

Monomers with antimicrobial properties have been proposed for the elaboration of photosensitive resins to be employed in stereolithography (SLA). An interesting example was reported by the group of Herrmann and Ren et al. [94], which developed a 3D printable polymeric resin based on monomers containing antimicrobial positively charged quaternary ammonium groups. Diurethanedimethacrylate/glycerol dimethacrylate (UDMA/GDMA) linear chains were photocured via light-induced polymerization. The antimicrobial dental parts were printed using a stereolithography set-up (Figure 8a). Interestingly, the printed parts kill bacteria on contact when positively charged quaternary ammonium groups are incorporated into the photocurable UDMA/GDMA resins. A post-functionalization process permits gradually quaternizing the samples as well as modifying the methacrylate monomers with different alkyl chain lengths (QA_C_n_). According to their results, the modification with an alkyl chain with a length of *n* = 12 presents the best antimicrobial performances (QA_C_12_). The contact killing efficacy was tested on Petri dishes by using *S. mutans* as a gram-positive model. A parallel, long-term antimicrobial effect was also measured after six days of culturing. The results demonstrated that the quaternization of the material considerably improved the killing rate of the printed parts. Finally, the printed parts were also mechanically tested (Figure 8b), showing performances quite similar to those of the other common 3D printed materials.

**Figure 9:**
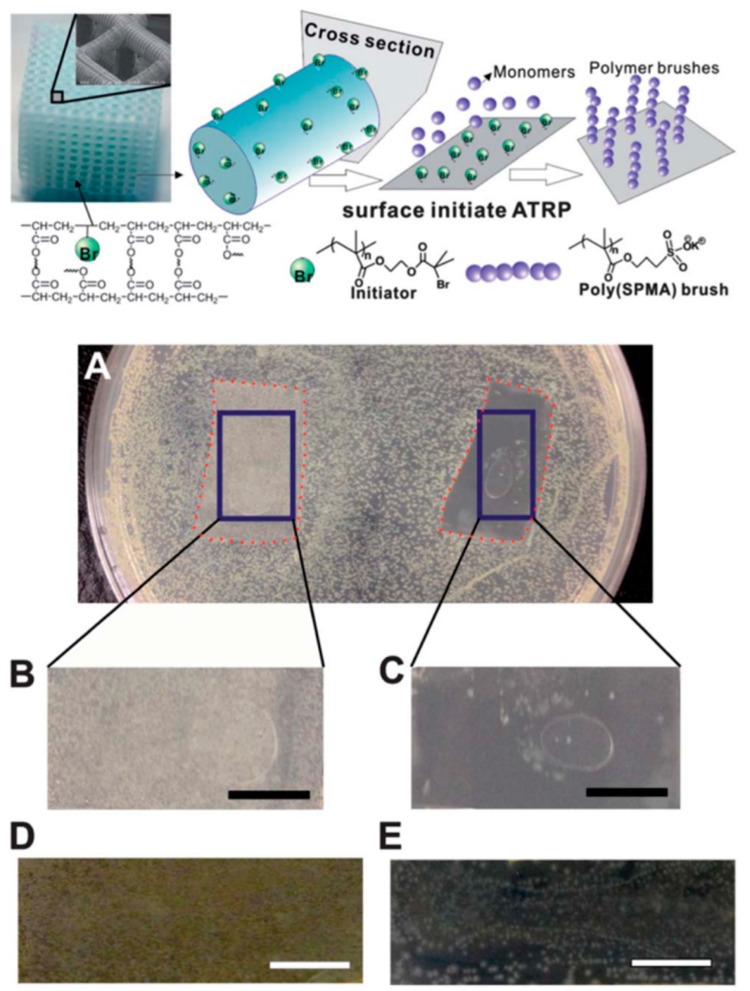
**Above** Schematic representation of the synthesis and grafting process of the 3D printed parts. **Below** (**a**) Photographs of the inhibition tests performed after 24 hours of bacteria growth, (**b**) the control sample and (**c**) 3-sulfopropyl methacrylate potassium salt (SPMA)-treated sample. Similar results after 48 hours for the (**d**) control and (**e**) SPMA-treated sample. Reproduced with permission from Ref. [5].

Similarly, the group of Yang and Wang et al. [5] performed some modifications to a commercial 3D printing resin called MiiCraft, which comprises acrylate-based pre-polymers, cross-linking agents, and a phosphine oxide-based compound as a photoinitiator (Figure 9a). Then, by using a stereolithography method, 3D printed parts were fabricated. In order to change the surface properties of the material, polymer brushes were grown on the printed structure via the surface-initiated atomic transfer data polymerization (SI-ATRP) grafting method. In this study, a thin layer of 3-sulfopropyl methacrylate potassium salt (SPMA) was grown on the printed polymer structure to bring antimicrobial properties to the printed part. Finally, bacterial adhesion and inhibition tests were carried out for the grafted samples. Both gram-negative (*E. coli*) and gram-positive (*B. subtilis*) bacteria were used as model microorganisms (Figure 9b). The results demonstrate that the functionalized surface can not only reduce the adhesion of the bacteria, but also inhibit the growth on the surface. The technique developed by Yang and Wang et al. has significantly expanded the capability of 3D printing technology for biomedical applications.

**Figure 10:**
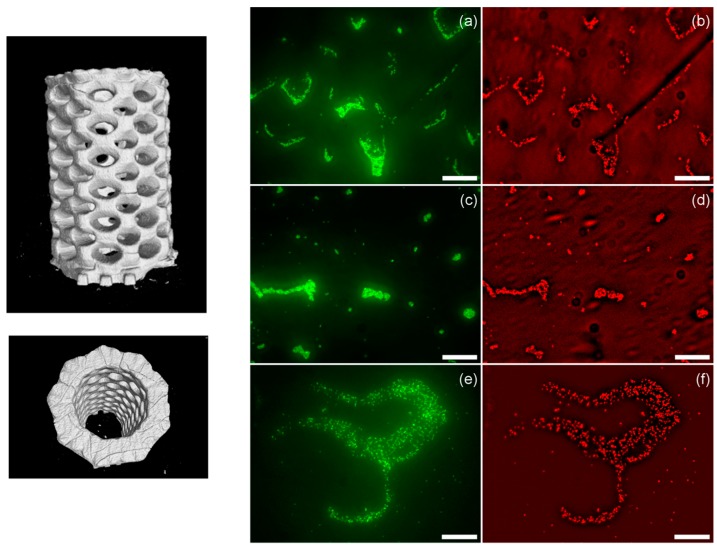
**Left:** Lateral and top 3D views of the micro-computed topography (µ-CT) images of the fabricated parts. **Right**: Bacterial viability on the different hydrogels with a variable amount of acrylic acid (AA). (**a**,**b**) 25 wt% AA, (**c**,**d**) 20 wt% AA, and (**e**,**f**) 10 wt% AA. Green fluorescence corresponds to the emission of all the bacteria, while the red one is related to the emission of propidium iodide (dead bacteria). Reproduced with permission from Ref. [95].

Another example was recently reported by Garcia et al. [95]. The group reported the preparation of both 3D printed pH-responsive and antimicrobial hydrogels with micrometric resolution using stereolithography. In particular, the authors prepared hydrogels using a resin formed by polyethylene glycol dimethacrylate (PEGDMA), with variable chain lengths (ranging from two up to 14 units) polyethylene glycol monomethacrylate, and acrylic acid (AA) as a linear monomer. Additionally, a photoabsorber (Sudan I) was employed in order to control the UV penetration depth. This allowed the authors to improve the printing model accuracy and simultaneously increase the z-axis resolution (Figure 7). Depending on the ratio between the components, a wide variety of smart hydrogels with variable swelling-responsive capacities were obtained. The groups reported that the hydrogel prepared by additive manufacturing was able to swell and shrink depending on the environmental pH. This effect is the consequence of the protonation and deprotonation of the carboxylic acid groups. Thus, the swelling observed is dependent on the amount of acrylic acid incorporated in the hydrogel formulation. Finally, as depicted in Figure 10, the hydrogels that were reported presented excellent antimicrobial properties for all of the compositions that were explored when exposed to S. aureus (bacteria employed as a model in this study).

### 7.2. Antimicrobial Polymers for FDM

**Figure 11:**
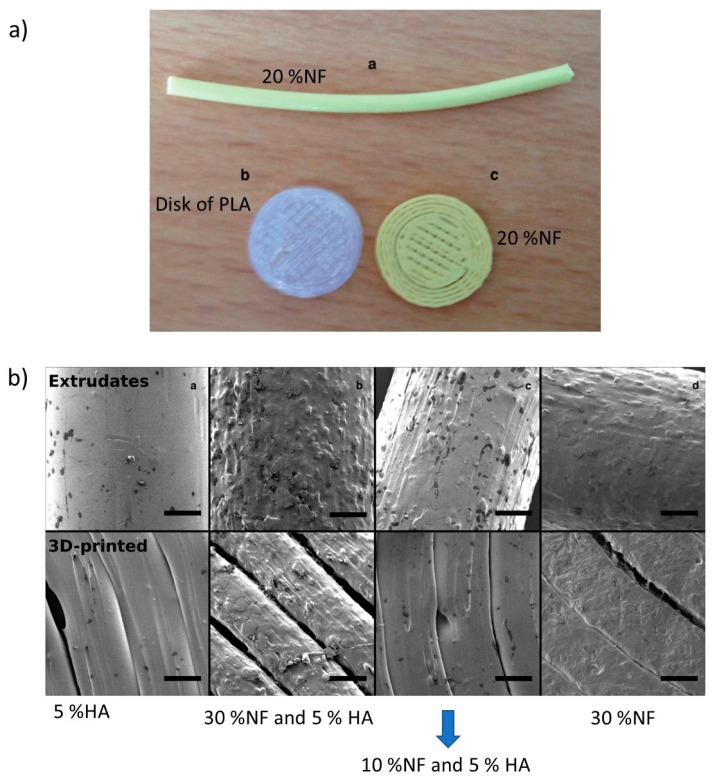
(**a**) Extrudated and 3D-printed and (**b**) SEM images of extrudated and 3D-printed geometries. Reproduced with permission from Ref. [96].

Antimicrobial thermoplastics or the elaboration of blends of thermoplastics with antimicrobial agents is a direct approach to prepare antimicrobial filaments that can be employed in FDM. For instance, Water et al. [96] characterized the physicochemical properties of the printed materials using thermoplastics charged with antimicrobial agents. More precisely, they used a custom-developed PLA feedstock material containing nitrofurantoin (NF) as an antimicrobial drug (10%, 20% or 30%) with and without the addition of 5% hydroxyapatite (HA) in polylactide strands. This mixture was used as a feedstock for the 3D printing process (Figure 11a). Figure 11b shows SEM images of the extrude and 3D materials where the surface morphology is highly dependent on the composition, which is to say, a sample with higher drug loading (30%) had an apparent rougher surface than those with lower drug content based on visual observations. The results indicated that the loading and release of NF from the printed PLA matrices showed an increased accumulated release after increasing the drug loading. Additionally, the materials showed resistance to bacterial adhesion and biofilm formation over a period of seven days. This research demonstrates the potential of custom-made, drug-loaded feedstock materials for the 3D printing of pharmaceutical products for controlled release. Similar studies that were carried out by Sandler et al. reported the possibility of incorporating NF in the PLA matrix in 5:95 (*w*/*w*) NF:PLA; this new material favored an 85% decrease of the biofilm formation on the 3D printed geometries over the 18-h time interval [8].

**Figure 12:**
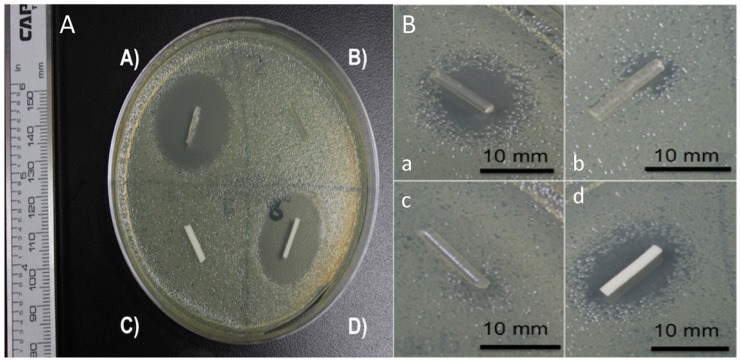
**A**. Gentamicin sulfate (GS)-doped poly(lactic acid) (PLA) and poly(methyl methacrylate) (PMMA) filaments. (**A**) 2.5 wt% gentamicin PLA filament; (**B**) Control PLA filament; (**C**) Control PMMA filament; (**D**) 2.5 wt% gentamicin PMMA filament. **B**. (**a**–**c**) 1 wt% gentamicin PLA filament; (**d**) 1 wt% gentamicin PMMA filament. Reproduced with permission from Ref. [97].

In a recent study, Mills et al. [97] reported the cytocompatibility, mechanical, and antimicrobial properties of 3D printed poly(methyl methacrylate) (PMMA) and poly(lactic acid) (PLA) beads loaded with a calculated amount of a drug in powdered form, i.e., gentamicin sulfate (GS). In this study, they showed that all the antibiotics that were studied were successfully doped into PMMA and in PLA, and antibiotic-doped 3D printed beads, disks, and filaments were easily printed by FDM. The growth inhibition capacity of the antibiotic-loaded PMMA 3D printed constructs was also demonstrated. The 1 wt% and 2.5 wt% gentamicin-doped PLA filaments and PMMA filaments were tested on bacterial plates (Figure 5). As depicted in Figure 12, those filaments without GS did not show any inhibition, whereas both PLA and PMMA presented an inhibition zone of around 23 mm.

**Figure 13:**
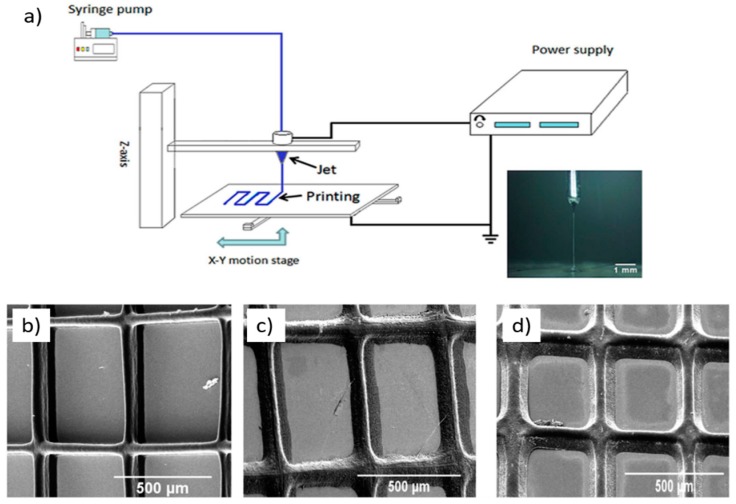
(**a**) Schematic description of the electrohydrodynamic (EHD) technique. SEM images of drug-loaded polycaprolactone (PCL)/polyvinyl pyrrolidone (PVP) patches at selected time intervals for the in vitro release study. (**b**) At 30 min; (**c**) At 60 min; and (**d**) At 90 min. Reproduced with permission from Ref. [42].

Another interesting example is that reported by the group of Wang et al. [42], who fabricated patches using AM technologies from polycaprolactone (PCL) and polyvinyl pyrrolidone (PVP). The parts also were charged with antibiotic drugs (tetracycline hydrochloride, TE-HCL) during the printing procedure. An interesting printing technique was used to fabricate the patches, the so-called electrohydrodynamic (EHD) technique, which is based on the application of an electric current through a nozzle that moves over a particular path to form a 3D printing part layer-by-layer (Figure 11a). This technique is very similar to electrospinning or the electrospray technique, but uses much lower voltages and currents to create the fibers. FTIR demonstrated successful TE-HCL encapsulation in the printed material. Patches prepared using PVP and TE-HCL displayed enhanced hydrophobicity compared to the rest. Also, uniaxial tensile mechanical tests were performed on the different materials; the printed parts exhibited changes in their mechanical properties arising from printing parameters such as fibrous strut orientation, variable inter-strut pore size, and film width. The release of antibiotics from PCL-PVP dosage forms was shown over five days, and was slower compared to pure PCL or PVP; also, the printed patch void size influenced antibiotic release behavior (Figure 13 b–d).

### 7.3. Surface Modification (Patterning and Functionalization) of 3D Printed Parts

An additional alternative to fabricating 3D printed parts with antimicrobial properties involves the surface modification of the part with antimicrobial agents. 

In this context, an illustrative strategy of this approach was recently reported by Vargas-Alfredo et al. [98] who reported a strategy that combines the use of high-impact polystyrene (HIPS) to fabricate 3D parts with surface functionalization methodologies to provide antimicrobial 3D objects. The scaffolds were first fabricated by FDM using commercially available HIPS filaments. Then, the object surface was modified, generating 3D parts with a particular strategy that permits simultaneously controlling the surface chemistry and the topography. In particular, the authors used the breath figures (BFs) approach. The BF strategy involves the scaffold immersion in a polymeric solution during a short (seconds) period of time. The immersion time was reported to be directly related to the pore size, and also help improve the bonding of the 3D printed layers. The results of the research showed evidence that the combination of BF and wet surface treatment is an interesting approach to control the surface microtopography (pore size) and surface functionality (a type of functional group and distribution) of 3D printed objects [98].

The 3D printed parts were fabricated via FDM using commercial polystyrene filament. The 3D porous structure was obtained via solvent evaporation from a polymeric solution: high molecular weight polystyrene (0 to 30 mg/mL) dissolved in chloroform, and water droplet condensation at the solvent/air interface occurring in a moist atmosphere. This procedure was carried out in a closed chamber with a saturated relative humidity at room temperature at different reaction time (one to five seconds). The results demonstrated that decreasing the polymeric solution produced a surface with smaller pores, which is an effect that is related to the viscosity of the reaction mixture. Additionally, a larger evaporation time finally produced pores with larger than average pore sizes. Subsequently, the surface chemical modification was carried out by immersion of the parts in the reactive chlorosulfonic acid solution at 20 °C at different reaction times (0 to 10 min).

**Figure 14:**
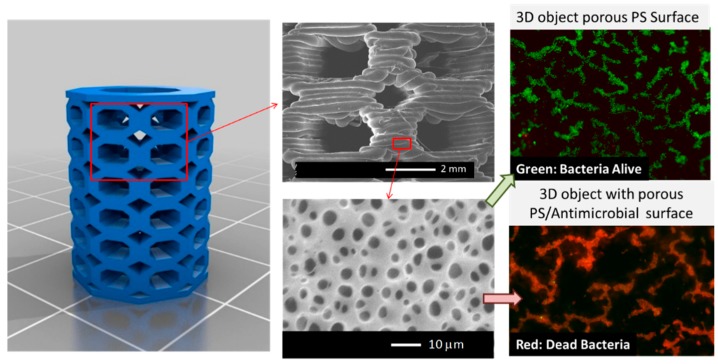
**Left:** 3D CAD model of scaffold selected for the fabrication by fused deposition modeling (FDM). **Middle:** SEM images of the scaffold surface revealing the layered structure obtained via FDM but also the formation of micropores. **Right:** Effect of the chemical composition on the antimicrobial properties of the 3D printed part. Above, without the antimicrobial functional group, and below, with the antimicrobial functional group. Reproduced with permission from Ref. [99].

More interestingly, by using polymer solutions comprising blends of PS and PS-*b*-PAA (PS_23_-*b*-PAA_18_) and a quaternized PS-*b*-poly(dimethylaminoethyl methacrylate) (PS_42_-*b*-PDMAEMA_17_), it was possible to simultaneously chemically modify the surface of the scaffold and therefore enable the incorporation of antimicrobial functional groups (Figure 14). Finally, the biological response of the surface-modified scaffolds against bacteria was investigated. The porous surfaces prepared using quaternized PDMAEMA as well as those prepared with PAA as the main component confer antimicrobial activity to the 3D printed parts, permitting killing *S. aureus* bacteria on contact. It should be mentioned that these functional supports are currently evaluated for the fabrication of functional devices such as biocompatible/antifouling tubes, screws for reparative surgery, or scaffolds in which the interactions’ cell support can be improved by finely tuning the surface properties (chemistry and structure) [99].

## 8. 3D Printed Polymeric Composites with Antibacterial Capacities

Finally, polymer composites have been explored also for the preparation of antimicrobial 3D printed parts. These composites have been fabricated using graphene [100] metals such as zinc, copper, or silver [87,101,102], and TiO_2_ [103] among others.

**Figure 15:**
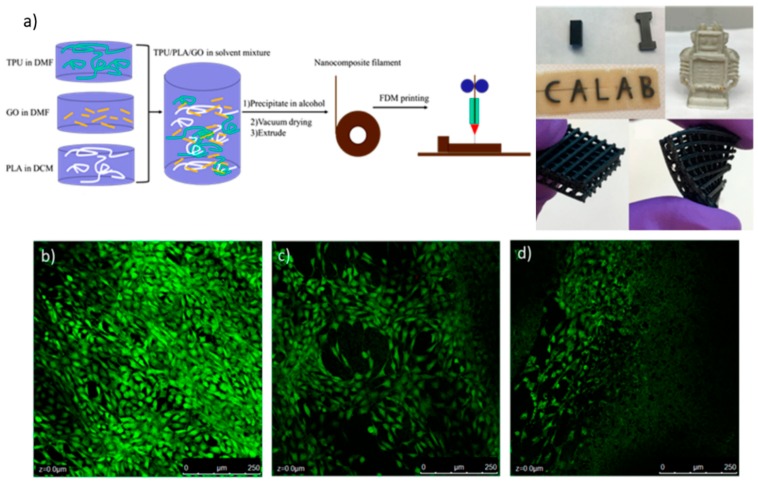
(**a**) Schematic description of FDM filament formation. Also, images of thermoplastic polyurethane (TPU)/poly(lactic acid) (PLA)/graphene oxide (GO) printed parts are shown. Green fluorescence images of cellular live/dead tests of the samples with different GO loads: (**b**) 0.5 wt%; (**c**) 2 wt%; and (**d**) 5 wt%. Reproduced with permission from Ref. [100].

A good example of this strategy was described by Advincula et al. [100], which blended a thermoplastic polyurethane (TPU), poly(lactic acid) (PLA), and graphene oxide (GO), which is a material that is known to be an excellent antimicrobial agent that could also enhance the mechanical properties of the final printed part. In this article, FDM methodology was used to print complex structures. To generate the FDM filament, TPU was dissolved in dimethylformamide (DMF), as well as GO nanocomposite; in parallel, PLA was dissolved in dichloromethane (DCM). Then, the three solutions were mixed under stirring overnight; then, the polymer–composite material was precipitated in alcohol and dried at 40 °C under vacuum. Finally, the resultant precipitate was melted in an extruder to form the FDM filament. According to the proportions of TPU and PLA, the material could have elastic characteristics, which were tested via mechanical tensile and compression experiments. The addition of GO has significantly enhanced the mechanical properties of the polymer matrix by 167% in a compression modulus, and 75.5% in a tensile modulus. Filaments with different GO loadings were tested as antimicrobial and biocompatible materials via live/dead studies. Figure 15 shows a scheme of FDM filament fabrication and some results from cell culture live/dead tests. The fluorescence images in Figure 15 demonstrate that none of the samples showed dead cells, which indicates a high level of material biocompatibility, thus demonstrating that the addition of GO has no obvious toxicity to cell growth, and a small amount of GO is beneficial for cell proliferation.

**Figure 16:**
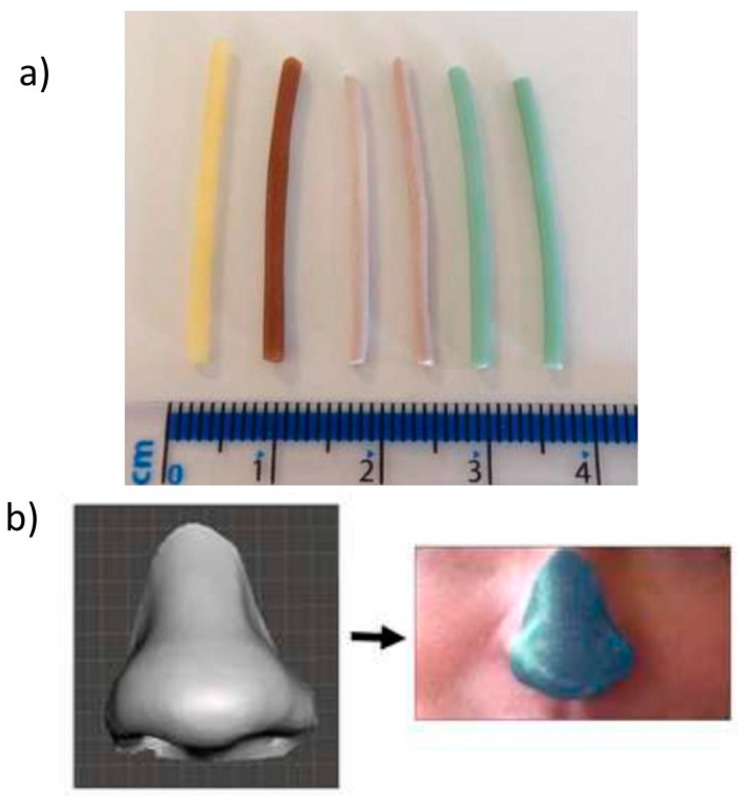
(**a**) Metal-loaded filaments and (**b**) 3D scan of a nose to create a designed wound dressing. Reproduced with permission from Ref. [87].

Muwaffak et al. [87] studied the use of antimicrobial metals such as zinc, copper, and silver incorporated into an FDA (Food and Drug Administration)-approved polymer (polycaprolactone, or PCL) to produce filaments for 3D printing (Figure 16a). 3D scanning was used to construct 3D models of a nose and ear to provide the opportunity to customize the shape and size of a wound dressing to an individual patient (Figure 16b). Experimentally, this research was based on the formation of a filament composed by different metal concentrations, whose release was studied by inductively coupled plasma atomic emission spectroscopy. Surface morphology and a cross-section of the filament were obtained by SEM analysis. Additionally, the possible bond of the metals with the PCL matrix was studied via FTIR spectra. The antibacterial efficacy of wound dressing was tested against *S. aureus*. To perform this type of study, a circular dressing was created using Tinkercard, which is a browser-based 3D design and modeling tool. According to these results, the silver and copper wound dressing had the most potent bactericidal properties.

**Figure 17:**
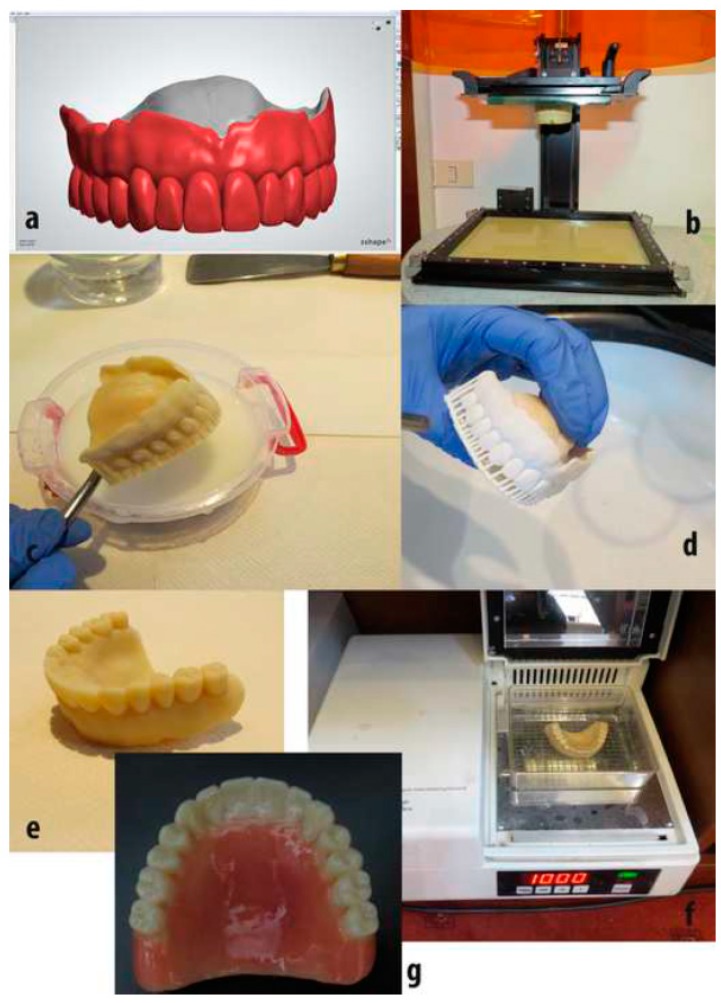
The protocol used to fabricate a complete denture, using the SLA printing technique. (**a**) Design in software. (**b**) Final construction in the 3D printer platform. (**c**) Denture cleaning with isopropanol. (**d**) Denture drying and supports removal. (**e**) Prototype denture polished. (**f**) Final post-curing procedure. (**g**) Esthetic adjustment. Reproduced with permission from Ref. [103].

Similarly, Cristache et al. [103] obtained a poly(methylmethacrylate) (PMMA)–TiO_2_ nanocomposite material with improved antibacterial characteristics, which was suitable for manufacturing 3D printed dental prosthesis. Different percentages of TiO_2_ nanoparticles (0.2, 0.4, 0.6, 1 and 2.5 weight %) were added gradually into PMMA solution. In parallel, the nanoparticles were synthesized through a sol–gel procedure from titanium tetrabutoxide, in the presence of dimedone, which was acting as a chelating agent, and dimethylacetamide. Structural and morphological analyses were carried out by FTIR, SEM, and Energy-Dispersive X-ray Spectroscopy (EDX), respectively. Finally, antimicrobial efficacy against bacterial cultures from *Candida species* (*C. Scotti*) was tested. The results proved that TiO_2_ nanoparticles modified the polymeric structure and their properties, especially the 0.4 % TiO_2_. This concentration was used to fabricate a prototype of a complete denture using the SLA printing method (Figure 17).

Similarly, Tiimob et al. [101] studied the effect of different proportions of eggshell/silver (ES-Ag) on the microstructure, thermal, tensile, and antimicrobial properties of 70/30 poly(butylene-*co*-adipate-terephthalate)/polylactic acid (PBAT/PLA). Additionally, the release kinetics of Ag nanoparticles (NPs) from the films was also studied. The thin films were prepared using hot melt extrusion and 3D printing for mechanical and antimicrobial testing. In vitro assessment of the antimicrobial activity of these films conducted on *L. monocytogenes* and *S. Enteritidis* bacteria at two different concentrations revealed that the blend composite film possessed bacteriostatic effects, which were due to the immobilized ES-Ag nanomaterials in the blend matrix. However, these were not released into distilled water or chicken breast after 72 h and 168 h of exposure, respectively. 

**Figure 18:**
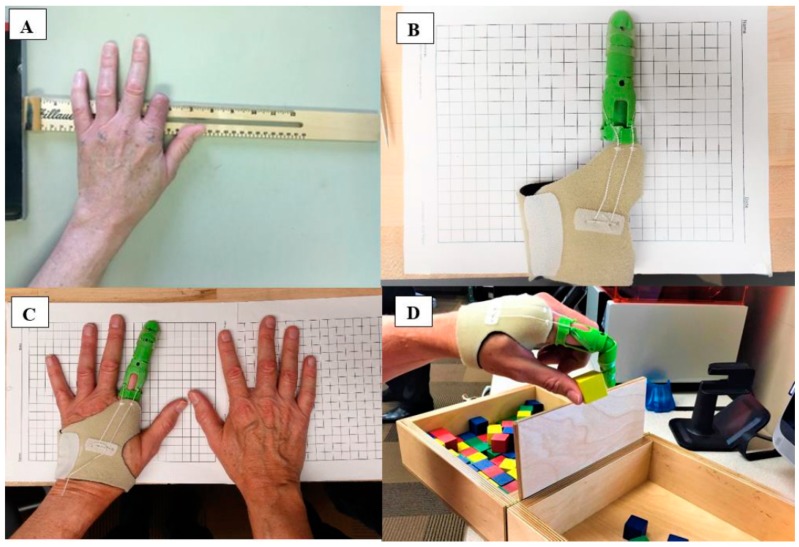
(**A**) Research participant with an index finger amputation. (**B**) 3D printer finder prothesis using PLACTIVE^TM^ antibacterial 3D filament. (**C**) Patient using the antibacterial 3D finger prosthesis; and (**D**) Patient performing the Box and Block Test. Reproduced with permission from Ref. [102].

Zuñiga [102] developed 3D printed prosthesis using antibacterial filaments, verifying their properties to kill certain bacteria. The author indicated that this information is relevant for the implementation of 3D printed prostheses as post-operative or transitional prostheses. Figure 18a shows a patient with a traumatic index finger at the proximal phalange. In Figure 18b,c, the patient was fitted with a 3D printed antibacterial finger prosthesis, and subsequently performed the Box and Block Test of manual gross dexterity (Figure 18d). *S. aureus* and *E. coli* were chosen to test the antibacterial capacity of the material, because these bacteria strains are the main causes of a variety of home-acquired and hospital-acquired infections. The prosthesis was performed using PLACTIVE^TM^ (1% antibacterial nanoparticles additive, Copper 3D, Santiago-Chile), which is a high-quality polylactic acid polymer. The main result of this research was to prove that the antibacterial 3D oriented filament can be effectively used for prostheses, and that their antibacterial properties after extrusion were not affected. 

The current investigation found that polylactic acid with 1% copper nanoparticles additives had up to 99.99% effectivity against *S. aureus* and *E. coli* after a 24-h incubation period. 

## 9. Conclusions

In this review, we attempted to provide a general overview about the state of the art in the preparation of antimicrobial objects fabricated by additive manufacturing. We described first the main principles of the currently employed AM technologies, as well as the evolution of these technologies, including the large impact that this technology has had in society. After this brief description, we discussed the main antimicrobial polymers, including the functional groups providing antimicrobial response as well as the macromolecular parameters involved in the antimicrobial response. We discussed the alternatives that have been reported to fabricate 3D antimicrobial parts, depending on the source of the polymers employed (natural or synthetic), as well as the strategy employed to introduce them in 3D printed parts (which were covalently linked if they acted as monomers in a photopolymerization, as additives, or simply by surface functionalization). Finally, the elaboration of composites that involve a polymeric matrix and charges including graphene or metals/metal oxide have been also reviewed.

3D printing technologies have enabled the preparation of personalized geometries with a wide variety of potential applications ranging from wound dressings, prosthesis, dental pieces, or complete denture. All of these biomedical applications without any doubt may benefit from the incorporation of antimicrobial polymers and additives that reduce the risk of infection by microorganisms. 

## Figures and Tables

**Table 1:** Summary of toxicity data available for the photoinitiators, photopolymers, and auxiliary compounds used in stereolithography (SLA). Reproduced with permission from ref [19].

Compounds	Available	Toxicological Information
**Photoinitiators**		
Phosphine oxide compounds ^1^	FORMlabs (Dental and E-Shell series)	Fertility-impairing effect [20], acute and chronic toxic for aquatic organisms [21], toxic effect on mouse NIH 3T3 cells [22]. Not readily biodegradable by Organization for Economic Cooperation and Development (OECD) criteria [21,23,24].
Benzophenone compounds ^2^	UV-cured inks	Causes liver hypertrophy and kidney adenoma in rats [25,26].
Triarylsulfonium salt (Cationic) ^3^	3D Systems	EC_50_ (24 h) Daphnia magna—4.4 mg/L [27]. EC_50_ (48 h) Daphnia magna—0.68 mg/L [27].
**Photopolymers**		
Acrylate monomers, Acrylate and Urethane acrylate oligomers	FORMlabs Autodesk Envisiontec 3D Systems	Toxic or harmful to various species of fish, algae, and water microorganisms [23]. Potential mutagens and a reproductive and developmental toxicant [28,29].
Methyl methacrylate monomers ^3^ and oligomers	FORMlabs (Envisiontec Dental resin)	Assessment of repeated dose toxicity indicates the potential to affect the liver and kidneys, as indicated in animal studies [30]. Potential mutagen, and a reproductive and developmental toxicant, aquatic toxicant, and genotoxic in mammalian cell culture [31,32,33].
Bisphenol A-diglycidyl dimethacrylate (Bis-GMA)	Dental resins	EC_50_ mouse fibroblast—9.35 μM [34].
**Auxiliary compounds**		
Butylated hydroxytoluene	Dental resins	Toxic or harmful to various species of fish, algae, and water microorganisms [35].
Sebacate compounds ^4^	FORMlabs (Dental Envisiontec)	Toxic to aquatic life with long-lasting effects [23], not readily biodegradable (OECD 301B) [23,36].
Hydroquinone	Dental resins	Evidence of mutagenicity in mammal studies, toxic to aquatic life; absorption, in sufficient concentrations, leads to cyanosis [37].

^1^ Including diphenyl(2,4,6-trimethylbenzoyl) phosphine oxide (TPO) and bis acyl phosphine oxide (BAPO). ^2^ Including benzophenone-3 (BP-3) and benzophenone-4 (BP-4) [38]. ^3^ Degrades to methacrylic acid: LD_50_ Oral rat—1320 mg/kg, LC_50_ (96 h) Oncorhynchus mykiss—85 mg/L [39]. ^4^ Bis(2,2,6,6-tetramethyl-4-piperidyl) sebacate, Pentamethyl-piperidyl sebacate.
